# Chronic methamphetamine uncovers a circadian rhythm in multiple-unit neural activity in the dorsal striatum which is independent of the suprachiasmatic nucleus

**DOI:** 10.1016/j.nbscr.2021.100070

**Published:** 2021-06-25

**Authors:** Shota Miyazaki, Yu Tahara, Christopher S. Colwell, Gene D. Block, Wataru Nakamura, Takahiro J. Nakamura

**Affiliations:** a Laboratory of Animal Physiology, School of Agriculture, Meiji University, Kawasaki, Kanagawa, 214-8571, Japan; b Department of Psychiatry and Biobehavioral Sciences, University of California Los Angeles, Los Angeles, CA, 90024-1759, USA; c Department of Electrical Engineering and Bioscience, School of Advanced Science and Engineering, Waseda University, Shinjuku, Tokyo, 162-8480, Japan; d Department of Biology, University of Virginia, Charlottesville, VA, 22904-4132, USA; e Department of Oral-Chrono Physiology, Graduate School of Biomedical Sciences, Nagasaki University, Nagasaki, Nagasaki, 852-8588, Japan

**Keywords:** Circadian rhythm, Dopamine, Methamphetamine, Multiple unit neural activity, Striatum, Suprachiasmatic nucleus, Wheel running activity, constant dark, (DD), dopamine, (DA), dopamine transporter, (DAT), light-dark, (LD), methamphetamine, (METH), multiple unit neural activity, (MUA), suprachiasmatic nucleus, (SCN), wheel running, (WR)

## Abstract

The dorsal striatum forms part of the basal ganglia circuit that is a major regulator of voluntary motor behavior. Dysfunction in this circuit is a critical factor in the pathology of neurological (Parkinson's and Huntington's disease) as well as psychiatric disorders. In this study, we employed *in vivo* real-time monitoring of multiple unit neural activity (MUA) in the dorsal striatum of freely moving mice. We demonstrate that the striatum exhibits robust diurnal and circadian rhythms in MUA that peak in the night. These rhythms are dependent upon the central circadian clock located in the suprachiasmatic nucleus (SCN) as lesions of this structure caused the loss of rhythmicity measured in the striatum. Nonetheless, chronic treatment of methamphetamine (METH) makes circadian rhythms appear in MUA recorded from the striatum of SCN-lesioned mice. These data demonstrate that the physiological properties of neurons in the dorsal striatum are regulated by the circadian system and that METH drives circadian rhythms in striatal physiology in the absence of the SCN. The finding of SCN-driven circadian rhythms in striatal physiology has important implications for an understanding of the temporal regulation of motor control as well as revealing how disease processes may disrupt this regulation.

## Highlights

• Dorsal striatum exhibits robust circadian rhythms in MUA in freely moving animals.
• Suprachiasmatic nucleus (SCN) lesions caused the loss of rhythmicity measured in the striatum.
• METH treatment made newly striatal MUA rhythms appear after SCN lesions.
• METH treatment reduced the amplitude and delayed the offset of SCN rhythms.

## 1 Introduction

In mammals, the generation of behavioral and physiological rhythms and the entrainment of these rhythms to the light-dark (LD) cycle are mediated by the suprachiasmatic nucleus (SCN) of the hypothalamus. It is known that the rhythms of locomotor activity, drinking behavior, and adrenal corticosterone are disrupted in SCN lesioned (SCNx) rodents (Stephan and Zucker, 1972; Moore and Eichler, 1972; Schwartz and Zimmerman, 1991; LeSauter and Silver, 1999). When rhythms of multiple unit neural activity (MUA) in the SCN are recorded by *in vivo* real-time monitoring, MUA is elevated during the subjective/objective day and depressed during the night, which is in antiphase with locomotor activity rhythms in nocturnal rodents (Kubota et al., 1981; Meijer et al., 1998; Yamazaki et al., 1998; Nakamura et al., 2008). The neural activity in the SCN is thought to drive rhythmic outputs that synchronizes circadian oscillators found throughout the body.

One of the clearest demonstrations of the multi-oscillator composition of the circadian timing system comes from experiments using methamphetamine (METH). When intact rodents were exposed to METH dissolved in drinking water, the free-running locomotor rhythm was lengthened in a way that was reversible after METH was removed from the drinking water (Honma et al., 1986; Mohawk et al., 2009; Tataroğlu et al., 2006). In some animals, the circadian organization was disturbed and two activity components appeared: one component was free-running (>24 h) and the other was entrained by the LD cycle (Honma et al., 1986; Tataroğlu et al., 2006). Importantly, when arrhythmic SCNx animals were treated with METH, robust rhythms in locomotor activity emerged (Tataroğlu et al., 2006; Honma et al., 1987, 1988). The METH regulation of locomotor activity rhythms does not necessary depend upon an intact molecular circadian clock as ultradian rhythms in locomotor activity are also lengthened in a variety of circadian clock gene mutants (Masubuchi et al., 2001; Mohawk et al., 2009; Pendergast et al., 2012; Blum et al., 2014). This data indicates that a METH-sensitive extra-SCN region can drive rhythms in locomotor activity.

The location of this METH sensitive regulator of rhythmic locomotor activity is not known but a wealth of data suggests that striatum is a likely substrate. First, the striatal circuit is critical for initiation and termination of movements (Prager and Plotkin, 2019; Grillner and El Manira, 2020). Basal dopamine (DA) levels or tone within the striatum are known to exhibit a daily rhythm with peaks during the night in nocturnal rodents (Hampp et al., 2008; Ferris et al., 2014). Acute administration of METH increases DA levels through dopamine transporter (DAT) inhibition while chronic treatments have complex effects that involve changes in the gene expression networks underlying DA signaling (Sulzer, 2011). For example, recent work describes a transcriptional reprograming in the striatum through dopamine D2R and PPARγ activation due to chronic cocaine treatment (Brami-Cherrier et al., 2020). Still the questions of whether the dorsal striatum exhibits circadian rhythms in neural activity and whether these rhythms are influenced by chronic METH exposure have not been addressed.

In the present study, we employed *in vivo* electrophysiological techniques to measure MUA rhythms in the dorsal striatum or SCN while simultaneously measuring wheel running (WR) rhythms in intact freely moving mice. We also recorded these circadian rhythms in mice that received an SCN lesion. Finally, we recorded these rhythms in mice that were exposed to chronic METH in their drinking water.

## 2 Materials and methods

### 2.1 Animals and housing

C57BL/6J male mice aged 3–5 months old were used for the experiments. Animals were maintained under controlled environmental conditions (temperature: 22 ± 2 °C; humidity: 50 ± 10%; light (L): dark (D) = 12:12 h; light: ~300 lux) with food and water available *ad libitum*. All procedures and standards of care were approved by the University of California, the Los Angeles Division of Laboratory Animals, the University of Virginia, the University of Virginia Institutional Animal Care and Use Committee, and the Institutional Animal Care and Use Committee at the School of Agriculture Meiji University. All procedures were conducted according to the National Institutes of Health guidelines for the use of experimental animals and the guidelines of the Japanese Physiological Society, respectively.

### 2.2 Measuring WR activity

For experiments assessing WR activity, each mouse was individually housed in a cage with a running wheel. The number of wheel revolutions was recorded in 1 min bins and analyzed with the Chronobiology Kit (Stanford Software Systems, Naalehu, HI, USA) or the ClockLab software (Actimetrics, Wilmette, IL, USA).

### 2.3 Producing SCNx mice

Experimental animals were anesthetized with isoflurane (2%–5%; Phoenix Pharmaceutical, Burlingame, CA, USA) and placed on a stereotaxic instrument (David Kopf Instruments, Tujunga, CA, USA). A Teflon-coated tungsten wire electrode (0.5 mm exposed tip) for the lesion was bilaterally inserted into the brain, aimed at the SCN (0.1 mm posterior and ±0.2 mm lateral to the bregma, 5.9 mm depth from the skull surface), and then a 1.1 mA DC current was applied for 20 s. We selected SCNx mice whose locomotor activities were arrhythmic in LD and constant darkness (DD) conditions. Following the study's completion, each animal was deeply anesthetized with isoflurane and rapidly decapitated. The brains were removed and fixed in 4% paraformaldehyde in 0.1 M phosphate buffer. Serial coronal sections (40 μm) were stained with neutral red, to confirm the successful SCNx.

### *2.4* *In vivo* real-time monitoring of MUA in the striatum and SCN

The experiments were performed as previously described (Nakamura et al., 2011). Experimental animals were set to the stereotaxic instrument as described above. Bipolar electrodes for the striatum were constructed from pairs of Teflon-coated stainless steel wires (bare diameter, 127 μm; Unique Medical, Tokyo, Japan) and an uncoated platinum-iridium wire (diameter, 75 μm; A-M Systems, Sequim, WA, USA). Bipolar electrodes for the SCN were constructed from pairs of Teflon-coated stainless steel wires (bare diameter, 75 μm; Unique Medical) and an uncoated platinum-iridium wire (diameter, 75 μm; A-M Systems). The electrodes were inserted into the brain, aimed at the striatum (0.1 mm anterior and 2.3 mm lateral to the bregma, and 3.0 mm depth from the skull surface) or the SCN (0.1 mm posterior and 0.2 mm lateral to the bregma, 5.9 mm depth from the skull surface) each, and attached directly to the skull with an orthodontic bond (3M, Saint Paul, MN, USA). After surgery, each mouse was transferred to an open-top cage with a running wheel mounted on one side, inside of a light-tight box, with a 12:12 h LD cycle. Output signals were processed by differential-input integration amplifiers (Burr-Brown, Tucson, AZ, USA) and then fed into AC amplifiers (bandpass, 500 Hz to 5 kHz; gain, × 10,000). Spikes were discriminated according to their amplitude and counted in 1 min bins using a computer-based window discrimination system. Simultaneously with the neural activity monitoring, the locomotor activity in individual mice was also detected as running wheel revolutions recorded in 1 min bins, which were then stored on a computer. Following the study's completion, each animal was deeply anesthetized with isoflurane, and a positive current (2 μA; 60 s) was passed through the recording electrodes. The brains were removed and fixed in 4% paraformaldehyde in 0.1 M phosphate buffer containing 5% potassium ferrocyanide (Sigma-Aldrich, St. Louis, MO, USA). Serial coronal sections (40 μm) were stained with neutral red, and blue spots of deposited iron were identified at the recording site.

### 2.5 Data analysis and statistics

The rhythm parameters were calculated using the ClockLab software. Free-running periods of MUA and WR were calculated by the chi-squared periodogram in 1 min bins for 6 days in DD condition (1st to 6th day in DD). The activity onset and offset of MUA were defined as the points where the activity exceeded and fell a standard mean activity for 3 days in the LD cycle in 1 min bins, respectively. The same definitions applied for the activity onset and offset of WR as well. The amplitude of MUA was defined as a subtraction of the peak-trough points, calculated by normalizing MUA for 3 days in the LD cycle to 24 h in 60 min bins. The statistical significance of the two different groups was determined by a Student's unpaired *t*-test. The statistical significances between different groups were determined by one-way analysis of variance (one-way ANOVA), followed by post-hoc Tukey tests. All results are presented as the means ± SEM and were considered significant at *p* < 0.05.

## 3 Experimental scheme

### 3.1 Experiment 1: *In vivo* MUA rhythms in the striatum of untreated SCN-intact (SCNi) mice and SCNx mice

Animals were randomly assigned into two groups, SCNi and SCNx, and housed in LD conditions for at least a week. The SCNx group underwent SCN lesion surgery. Bipolar electrodes were inserted into the striatum for the MUA recordings. After 1 week of recovery from the surgery, the MUA and WR activities were simultaneously recorded in LD and DD conditions. Food and drinking water were available *ad libitum* (Fig. S1A).

### 3.2 Experiment 2: *In vivo* MUA rhythms in the striatum of METH-treated mice

Animals were randomly assigned into two groups: SCNi with METH and SCNx with METH. The SCNx group received SCN lesions as described above, while SCNi group received a sham surgery. After recovery, all experimental animals were treated with 0.005% METH dissolved in their drinking water until they displayed a METH-induced circadian locomotor activity rhythm in DD condition. Based on the previous report (Tataroğlu et al., 2006), a METH-induced circadian locomotor activity rhythm was defined as whether or not the period exceeded 24 h calculated by the chi-squared periodgram. After each mouse displayed the correct rhythmicity, bipolar electrodes were inserted into the striatum to carry out the MUA recordings. After 1 week of recovery from surgery, MUA and WR activities were simultaneously recorded in DD condition for at least 6 days. Food and 0.005% METH dissolved in drinking water were available *ad libitum* (Fig. S1B).

### 3.3 Experiment 3: *In vivo* MUA rhythms in the SCN of METH-treated mice

Animals were randomly assigned into two groups: control (Cont) and METH-treated. As in Experiment 2, METH-treated animals were housed in DD condition and treated with 0.005% METH dissolved in drinking water until they displayed a METH-induced circadian locomotor activity rhythm while Cont animals were treated with normal drinking water. Based on the previous report (Tataroğlu et al., 2006), a METH-induced circadian locomotor activity rhythm was defined as whether or not the period exceeded 24 h calculated by chi-squared periodgram. When each mouse displayed the rhythmicity, bipolar electrodes were inserted into the SCN in order to carry out the MUA recordings. After 1 week of recovery from surgery, MUA and WR activities were simultaneously recorded in LD and DD conditions. Food and each experimental drinking water were available *ad libitum* (Fig. S1C).

## 4 Results

### 4.1 The MUA rhythm in the striatum reflects WR activity in untreated SCNi mice, and these rhythms are lost in SCNx mice

**Fig. 1:**
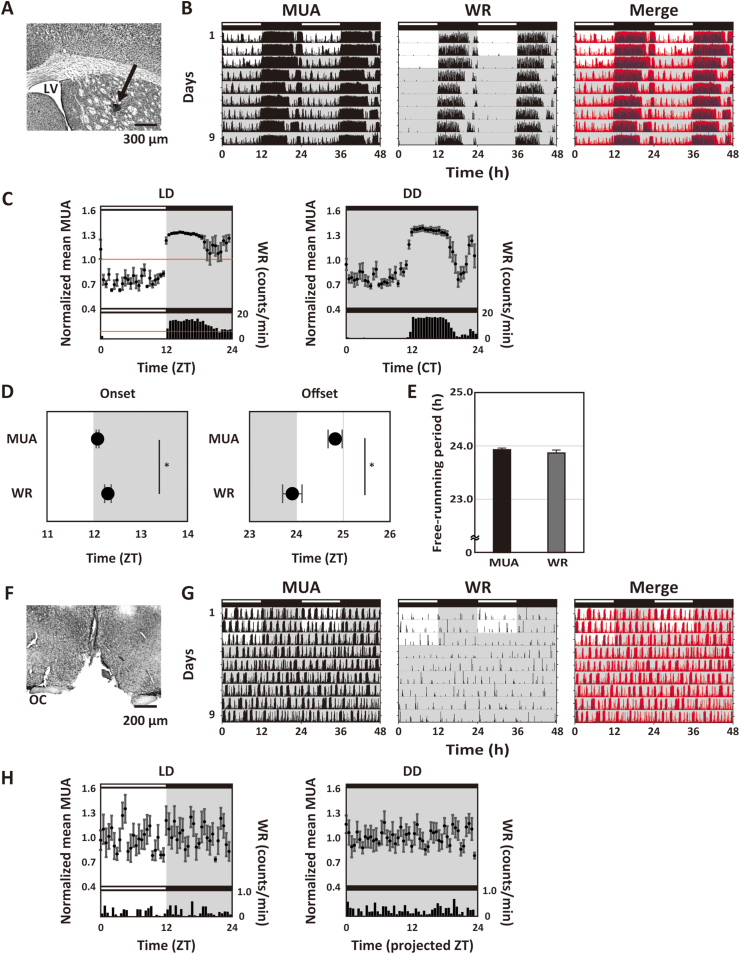
Simultaneous recording of MUA in the striatum and WR rhythms in freely moving SCNi and SCNx mice. (A) A typical histological section indicates the recording site with a neutral red stain. The black arrowhead indicates the recording site in the striatum. The scale bar is 300 μm. (B) Representative double-plotted actograms showing diurnal and circadian rhythms of MUA in the striatum, WR and merged in SCNi mice. The red and blue actograms indicate MUA and WR, respectively. The mice were maintained in the LD cycle and then transferred into the DD condition. Lighting conditions are indicated at the top of the figure; open bars indicate a light phase and closed indicate a dark phase. (C) Representative integrated mean activities of MUA and WR in SCNi mice were plotted according to LD cycle and DD condition. MUA in the LD cycle for 3 days was normalized to 24 h in 30 min bins; each point indicates a level of MUA counts at the time relative to average MUA counts at 24 h. WR in the LD cycle for 3 days was plotted in 60 min bins. Red line indicates the standard mean activity of MUA and WR. As in the LD cycle, each MUA and WR in the DD condition for 6 days were also plotted to CT. Data were shown as means ± SEM in 30 min bins (MUA) or 60 min bins (WR). (D) The activity onsets and offsets of MUA in the striatum and WR in SCNi mice were calculated under the LD cycle. (E) The free-running periods of MUA in the striatum and WR in SCNi mice were calculated. *p < 0.05, Student's unpaired *t*-test. (F) A typical histological section indicates that the SCN was lesioned by a positive current. The scale bar is 200 μm. (G) Representative double-plotted actograms showing the diurnal and circadian rhythms of MUA in the striatum, WR, and merged in SCNx mice. The red and blue actograms indicate MUA and WR, respectively. The mice were maintained in the LD cycle and then transferred into the DD condition. The lighting conditions are indicated at the top of the figure; open bars indicate light phase and closed indicate dark. (H) Representative integrated mean activities of MUA and WR in SCNx mice plotted in LD and DD. Each MUA and WR in LD for 3 days were normalized to 24 h. Each MUA and WR in DD for 6 days were normalized to 24 h. . (For interpretation of the references to colour in this figure legend, the reader is referred to the Web version of this article.)

**Fig. 2:**
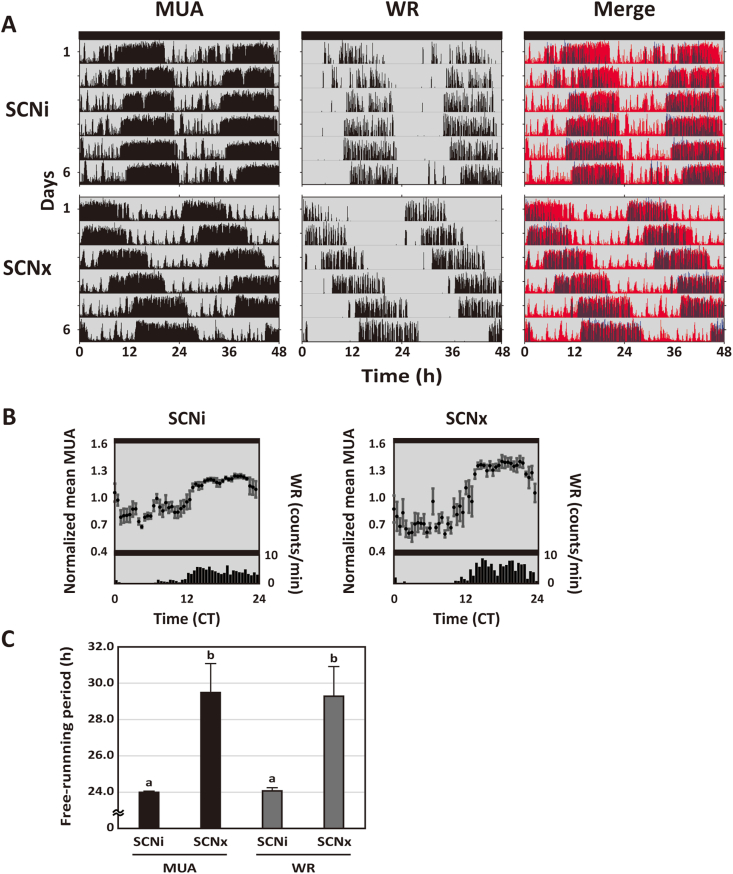
*In vivo* MUA rhythms in the striatum of METH-treated SCNi and SCNx mice. (A) Representative double-plotted actograms showing circadian rhythms of MUA in the striatum, WR and merged in METH-treated SCNi and SCNx mice. The red and blue actograms indicate MUA and WR, respectively. The actograms represent DD conditions for 6 days. (B) Representative integrated mean activities of MUA and WR plotted under the DD condition. MUA in the DD condition for 6 days was normalized to a circadian time in 30 min bins; each point indicates a level of MUA counts at the time relative to average MUA counts at a circadian time. WR in the DD condition for 6 days was plotted in 60 min bins to a circadian time. Data were shown as means ± SEM in 30 min bins (MUA) or 60 min bins (WR). (C) The free-running periods of MUA in the striatum and WR in METH-treated SCNi and SCNx mice were calculated. Differing letters between groups indicate significant differences (*p* < 0.05, Tukey's test). (For interpretation of the references to colour in this figure legend, the reader is referred to the Web version of this article.)

We first implanted a single bipolar electrode into the striatum to monitor the MUA (Fig. 1A). The rhythms of MUA in the striatum and WR were simultaneously recorded in SCNi mice. The MUA exhibited robust diurnal and circadian rhythms, which were active during the night, in phase with WR (Fig. 1B and C, S2A, and S5A). The onset of the MUA rhythms was earlier than that of the WR rhythms (Fig. 1D; MUA onset: 12.08 ± 0.03 h; WR onset: 12.30 ± 0.07 h; Student's unpaired *t*-test: *p* = 0.026; n = 4), whereas the offset of the MUA rhythms was later than that of the WR rhythms (MUA offset: 24.83 ± 0.15 h; WR offset: 23.91 ± 0.21 h; Student's unpaired *t*-test: *p* = 0.012; n = 4). When the MUAs in the striatum were higher level, the WR levels were also high. However, the two rhythms were not completely parallel with each other (Fig. S3). The free-running periods of the MUA rhythms were indistinguishable from those of the WR rhythms (Fig. 2E; MUA: 23.93 ± 0.03 h; WR: 23.87 ± 0.05 h; Student's unpaired *t*-test: *p* = 0.47; n = 4). These data indicate that the neural activity of the striatum exhibits robust diurnal and circadian rhythms and that the phasing of these rhythms parallel WR activity. We also recorded the MUA in the striatum and WR rhythms in SCNx mice (Fig. 1F). Fig. 1G shows the double-plotted actogram for the MUA and WR under LD and DD conditions. Both circadian rhythms disappeared following the SCNx surgery (Fig. 1H and S2B), while it seemed that the activity pattern of striatal MUA and WR were similar in SCNx mice (Fig. 1G and S5B). These data indicate that the circadian rhythms of MUA in the striatum and WR are dependent upon the intact SCN under normal conditions.

### 4.2 METH affects striatal MUA rhythms in SCNi mice and makes newly circadian oscillations of striatal MUA appear in SCNx mice

We next examined the effects of METH on the MUA in the striatum of SCNi and SCNx mice. All experimental SCNi and SCNx mice showed METH-induced circadian locomotor activity rhythms in DD condition before surgery for MUA recording (Fig. S4). We implanted single bipolar electrodes into the striatum and recorded the MUA when the mice were held in DD conditions. The MUA in both the METH-treated mice groups exhibited robust circadian rhythms that were in phase with the METH-induced circadian locomotor activity rhythms (Fig. 2A, B, S2C, S2D, S5C, and S5D). The free-running periods of the MUA rhythms were indistinguishable from those of the WR rhythms in both SCNi and SCNx mice. However, the free-running periods of the MUA and WR rhythms in SCNx mice were longer than those in SCNi mice (Fig. 2C; MUA in SCNi mice: 23.99 ± 0.07 h; MUA in SCNx mice: 29.49 ± 1.59 h; WR in SCNi mice: 24.07 ± 0.18 h; WR in SCNx mice: 29.28 ± 1.64 h; one-way ANOVA, a post-hoc Tukey test: *p* < 0.05; n = 4, respectively). These data indicate that METH strongly affects the neural activity rhythms in the striatum of SCNi mice and, importantly, METH treatment undercovers the expression of a circadian rhythm of MUA in the striatum of SCNx mice.

### 4.3 METH has some impact on MUA recorded in the SCN

**Fig. 3:**
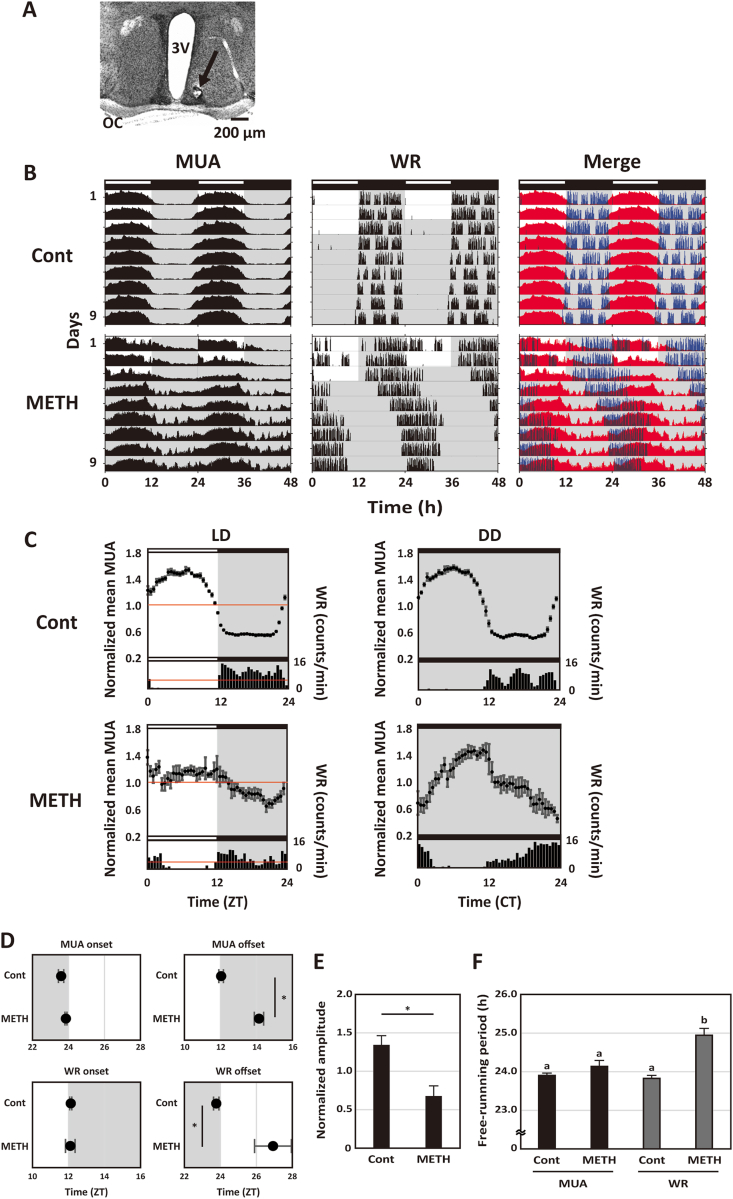
*In vivo* MUA rhythms in the SCN of METH-treated mice. (A) A typical histological section indicates the recording site using a neutral red stain. The black arrowhead indicates the recording site in the SCN. The scale bar indicates 200 μm. (B) Representative actograms showing diurnal and circadian rhythms of MUA in the SCN, WR and merged in METH-untreated (Cont) and METH-treated (METH) mice. The red and blue actograms indicate MUA and WR, respectively. The mice were maintained in the LD cycle and then transferred into the DD condition. The lighting conditions are indicated at the top of the figure; open bars indicate light phase and closed indicate dark. (C) Representative integrated mean activities of MUA and WR in Cont and METH-treated mice were plotted according to LD cycle and DD condition. MUA in the LD cycle for 3 days was normalized to 24 h in 30 min bins; each point indicates a level of MUA counts at the time relative to average MUA counts at 24 h. WR in the LD cycle for 3 days was plotted in 60 min bins. Red line indicates the standard mean activity of MUA and WR. As in the LD cycle, each MUA and WR in the DD condition for 6 days were also plotted to CT. Data were shown as means ± SEM in 30 min bins (MUA) or 60 min bins (WR). (D) The activity onsets and offsets of MUA in the SCN and WR in Cont and METH-treated mice were calculated in the LD cycle, respectively. *p < 0.05, Student's unpaired *t*-test. (E) The amplitude of MUA in the SCN in Cont and METH-treated mice were calculated in the LD cycle. *p < 0.05, Student's unpaired *t*-test. (F) The free-running periods of MUA in the SCN and WR in Cont and METH-treated mice were calculated. Differing letters between groups indicate significant differences (*p* < 0.05, Tukey's test). (For interpretation of the references to colour in this figure legend, the reader is referred to the Web version of this article.)

Finally, we examined the effects of METH on the MUA recorded in the SCN. We implanted single bipolar electrodes into the SCN to monitor the MUA (Fig. 3A). MUA in untreated (Cont) mice exhibited robust diurnal and circadian rhythms, with most of the activity occurring during the day, in antiphase with WR (Fig. 3B, S2E, S5E, and S5F). When treated with METH, mice still displayed circadian locomotor activity and exhibited robust rhythms of MUA in the SCN (Fig. 3B, C, and S2F). METH did not affect the onsets of MUA in the SCN nor WR rhythms (Fig. 3D; MUA onset in Cont mice: 23.59 ± 0.15 h; MUA onset in METH-treated mice: 23.86 ± 0.06 h; Student's unpaired *t*-test: *p* = 0.20; Cont: n = 4, METH: n = 3; WR onset in Cont mice: 12.12 ± 0.06 h; WR onset in METH-treated mice: 12.10 ± 0.27 h; Student's unpaired *t*-test: *p* = 0.93; Cont: n = 4, METH: n = 3). However, METH dramatically reduced the amplitude of the MUA rhythms recorded in the SCN (Fig. 3E; the amplitude in Cont mice: 1.34 ± 0.14; the amplitude in METH-treated mice: 0.68 ± 0.16; Student's unpaired *t*-test: *p* = 0.025; Cont: n = 4, METH: n = 3). In addition, METH delayed the offsets of MUA rhythms in the SCN and WR rhythms (Fig. 3D; MUA offset in Cont mice: 12.04 ± 0.13 h; MUA offset in METH-treated mice: 14.14 ± 0.26 h; Student's unpaired *t*-test: *p* = 0.00051; Cont: n = 4, METH: n = 3; WR offset in Cont mice: 23.77 ± 0.15 h; WR offset in METH-treated mice: 26.92 ± 1.02 h; Student's unpaired *t*-test: *p* = 0.015; Cont: n = 4, METH: n = 3). Interestingly, METH only lengthened the free-running periods of the WR rhythms (Fig. 3F; MUA in Cont mice: 23.90 ± 0.06 h; MUA in METH-treated mice: 24.14 ± 0.16 h; WR in Cont mice: 23.83 ± 0.08 h; WR in METH-treated mice: 24.95 ± 0.18 h; one-way ANOVA, a post-hoc Tukey test: *p* < 0.01; n = 3, respectively). These data indicate that METH strongly affects the WR rhythms but not the period of the neural activity in the SCN.

## 5 Discussion

In this study, we recorded robust diurnal and circadian rhythms in electrical activity in the striatum that peaked during the night and broadly mirrored the rhythms that we measured in WR activity (Fig. 1). The MUA in the striatum overlapped WR activity bouts in a short scale (Fig. 1D and S3). *In vivo* microdialysis sampling in the striatum and high-performance liquid chromatography analysis has revealed that the striatum contains monoamine neurotransmitters and are innervated by dopaminergic neurons (Stiles et al., 2018). It is also known that dopaminergic signals into the striatum regulate behaviors such as locomotion and motivation (Mendoza and Challet, 2014; De Corte et al., 2019). Therefore, it is reasonable that the striatal neural activity is closely related to the WR behavior in mice. Both circadian behavioral rhythm and MUA rhythm in the striatum were observed in SCNi mice, while the circadian rhythms disappeared in SCNx mice. Taken together, it is suggested that the MUA in the striatum and WR are related to each other *in vivo*, and the circadian rhythmicity are dependent upon the SCN and are lost with SCNx.

In the present study, we also showed that treatment with METH in the drinking water made the rhythms in neural activity in the striatum appear recorded in SCN-lesioned mice (Fig. 2). This data compliments long standing observations that METH can drive rhythms locomotor behavior in SCN-lesioned animals (Honma et al., 1987; Tataroğlu et al., 2006). The mechanisms underlying the METH sensitive oscillator are still unclear but these oscillatory mechanisms do not require circadian clock genes (Masubuchi et al., 2001; Mohawk et al., 2009; Pendergast et al., 2012; Blum et al., 2014). This later work from Storch and colleagues explored the *Bmal1* KO mice and found evidence for an arousal regulating ultradian oscillator that exhibits a wide range of cycle lengths depending upon the concentration of METH to which the mouse was exposed (Blum et al., 2014). Still it is worth emphasizing that we do know the mechanism by which the application of low concentrations of METH in the drinking water cause these dramatic effects. Acute administration of METH increases levels of DA through competitive DAT inhibition (e.g. Giros et al., 1996; Prakash et al., 2017) but the chronic impact of these stimulants is much less clear. A recent study looking at the effects of cocaine found that chronic application caused major changes in circadian gene expression within the striatum (Brami-Cherrier et al., 2020) including the induction of new oscillations in PPARγ target genes. There is every reason to think that this type of complexity is also occurring in our mice treated with METH.

Finally, we found that METH altered the MUA rhythms recorded in the SCN, although the SCN fluctuated itself under METH exposure (Fig. 3). The results are consistent with the possibility of internal desynchronization between two oscillators, as shown by locomotor analysis in METH-treated animals (Tataroğlu et al., 2006). Specifically, chronic METH treatment reduced the amplitude (Fig. 3E) and delayed the offset (Fig. 3D) of the MUA rhythm without changing the onset of the rhythm. Prior analysis of SCN-intact rodents has repeatedly shown that chronic METH dramatically lengthens the circadian period of locomotor activity rhythms (e.g. Honma et al., 1986; Tataroğlu et al., 2006; Mohawk et al., 2009) but this same treatment does not appear to alter rhythms in clock gene expression measured in the SCN (Masubuchi et al., 2000). These observations lead Pendergast and colleagues to propose a working model in which METH-exposure induces a robust extra-SCN oscillator which inhibits SCN output (Pendergast et al., 2013). Our electrophysiological data fits extremely well with this model as we demonstrate that the rhythmic electrical output of the SCN is reduced under METH (Fig. 3E). We do not know how METH is altering the SCN output. DA signaling pathways mediated by DA receptor type 1 (DR1) are active within the SCN where DR1 regulates entrainment to light (Grippo et al., 2017) as well as metabolic processes (Grippo et al., 2020). Thus, the most parsimonious explanation is that chronic METH alters DA signaling within the SCN to reduce the neural activity rhythms but this remains to be confirmed. On the other hand, a METH-induced circadian locomotor activity feeds back to the SCN (Honma et al., 1991) and the feedback of normal wheel running activity alters the SCN periodicity (Yamada et al., 1988). We confirmed some impacts on rhythm parameters in the SCN in METH-treated mice in this study (Fig. 3D and E), which could be explained in the context of feedback from locomotor activity. To confirm that the SCN is affected by whether the direct effects of METH or something that METH induced *in vivo* (e.g. a METH-induced circadian oscillator, WR feedbacks), additional experiments will be required.

In conclusion, we recorded MUA rhythms in the striatum and SCN using *in vivo* real-time monitoring. Our data demonstrate that the dorsal striatum expresses a circadian rhythm in MUA that is normally under the control of the SCN. Chronic METH treatment uncovers a SCN-independent oscillation in MUA that we recorded in the striatum. Chronic METH treatment also reduced the amplitude and delayed the offset of the MUA rhythms recorded from the SCN. These measurements support the view that chronic treatment with stimulants can reprogram circadian transcription within the striatum (Masubuchi et al., 2001; Brami-Cherrier et al., 2020). However, our results are based on a limited number of data and the conclusion should be viewed as tentative until more data can be collected. For example, we have to consider aftereffects of prior entrainment in their circadian periods due to the calculation for 6 days after switching to DD in all treatment groups. Thus, more long-term recording in DD is needed to exclude the aftereffects of light entrainment. Moreover, the work described in the present study does not identify the mechanism of how METH affected the SCN rhythmicity. Additional experiments would be required to clearly establish the pathway regulated by METH. Given the central role of the dorsal striatum in the control of movements as well as the pathology of neurological disorders that impact movement, the circadian reprogramming driven by chronic treatment with dopamine receptor agonists is an important area for future work.

## CRediT authorship contribution statement

**Shota Miyazaki:** performed the experiments, Formal analysis, Writing – original draft. **Yu Tahara:** performed the experiments, Formal analysis, wrote the paper. **Christopher S. Colwell:** Methodology, Writing – original draft. **Gene D. Block:** Methodology. **Wataru Nakamura:** Methodology, designed the research. **Takahiro J. Nakamura:** Formal analysis, Writing – original draft.

## Declaration of competing interest

None.
