# Supplementary material for: Chronic methamphetamine uncovers a circadian rhythm in multiple-unit neural activity in the dorsal striatum which is independent of the suprachiasmatic nucleus
Source: Neurobiol Sleep Circadian Rhythms. 2021 Jun 25;11:100070. doi: 10.1016/j.nbscr.2021.100070 (PMC8258683; doi:10.1016/j.nbscr.2021.100070)
Supplement: Multimedia component 1 [file mmc1.docx]

**Supplementary Information for**

**Chronic methamphetamine uncovers a circadian rhythm in multiple-unit neural activity in the dorsal striatum which is independent of the suprachiasmatic nucleus**

**Shota Miyazaki, Yu Tahara, Christopher S. Colwell, Gene D. Block, Wataru Nakamura, Takahiro J. Nakamura**

**This PDF file includes:**

**Figures S1 to S5**


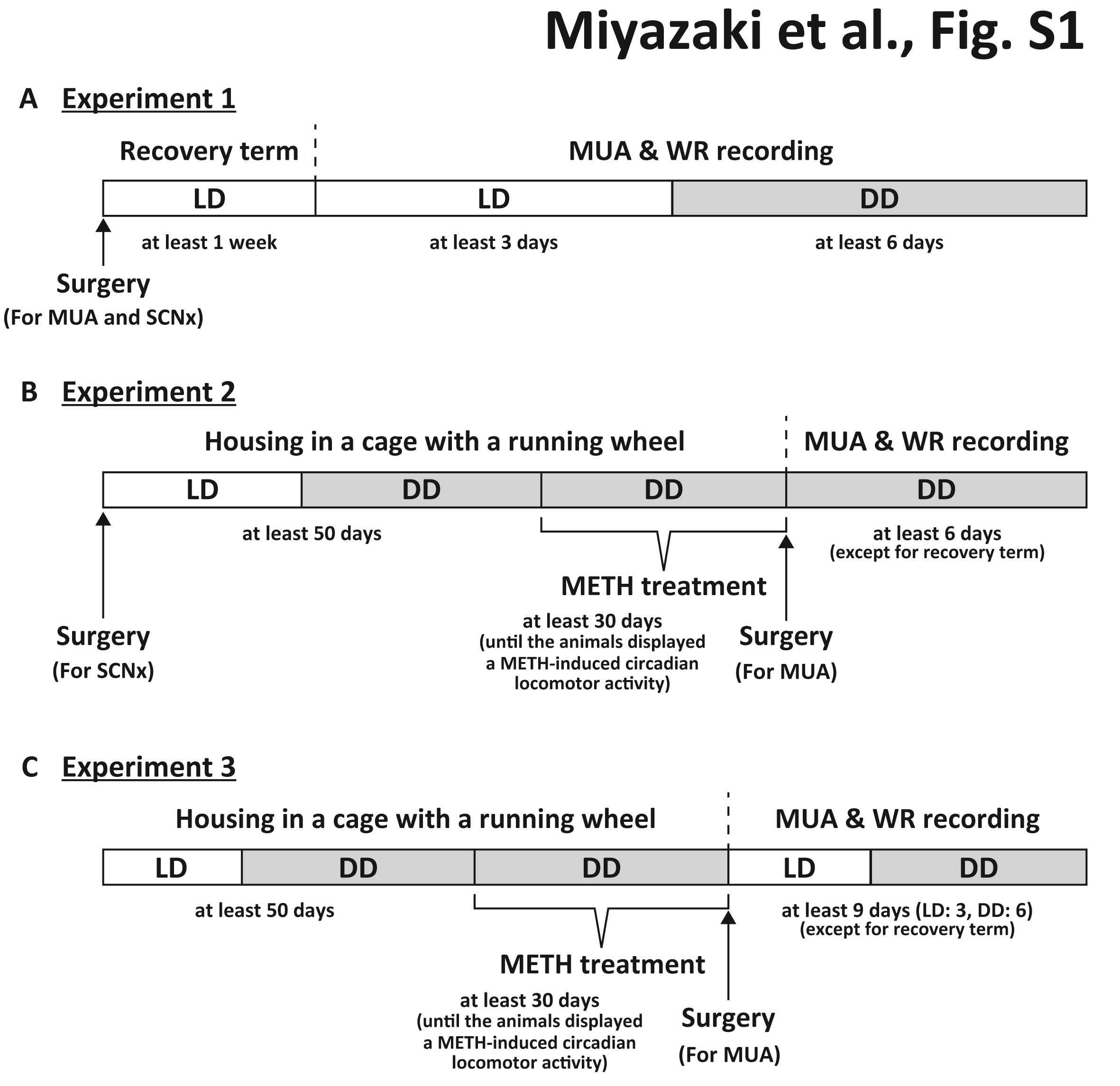


**Fig. S1 Flow chart of experimental scheme in this study.**

(A) The model showing a scheme in Experiment 1. Experimental mice were recovered for at least 1 week after a surgery for MUA recording. In SCNx mice, a SCNx surgery was also performed at the same time. MUA and WR activities were simultaneously recorded in the LD cycle for at least 3 days and in DD condition for at least 6 days. (B) The model showing a scheme in Experiment 2. At first, the mice received a SCNx surgery or a sham surgery. After that, the mice were singly housed in a cage with running wheel in the LD cycle and DD condition for at least 50 days, followed by treatment with 0.005 % METH dissolved in drinking water for at least 30 days. After each mouse displayed a METH-induced circadian locomotor activity rhythm, a surgery for MUA recording was performed. Animals in DD were returned to LD for MUA electrode implantation and returned to DD after 1week of recovery. After that, MUA and WR activities were simultaneously recorded in DD condition for at least 6 days. (C) The model showing a scheme in Experiment 3. The mice were singly housed in a cage with running wheel in the LD cycle and DD condition for at least 50 days. After that, only experimental animals projected to METH-treated mice were exposure to 0.005 % METH dissolved in drinking water for at least 30 days. After each mouse displayed the rhythmicity, a surgery for MUA recording was performed. After 1 week of recovery from surgery in LD, MUA and WR activities were simultaneously recorded in the LD cycle for at least 3 days and in DD condition for at least 6 days.


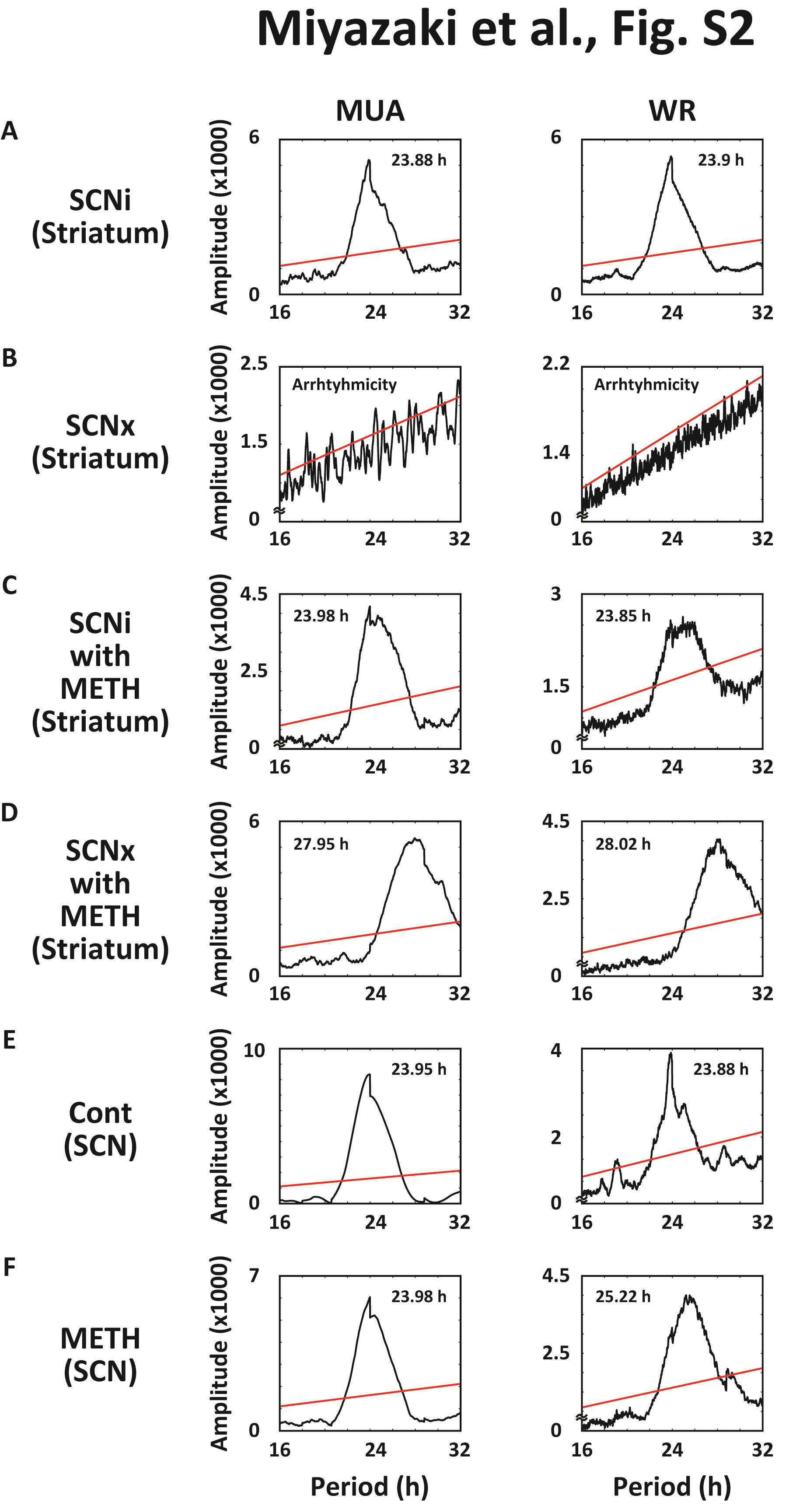


**Fig. S2 The periodogram analysis of MUA and WR rhythms in each representative experimental mouse.**

The chi-squared periodgram of MUA and WR rhythms for 6 days in DD condition in SCNi (A) and SCNx (B) mice in Fig. 2, SCNi with METH (C) and SCNx with METH (D) mice in Fig. 3, and Cont (E) and METH-treated (F) mice in Fig. 4. The oblique red line indicates the significance level (**p* < 0.001).

**Fig. S3 MUA and WR activities in a short scale.**


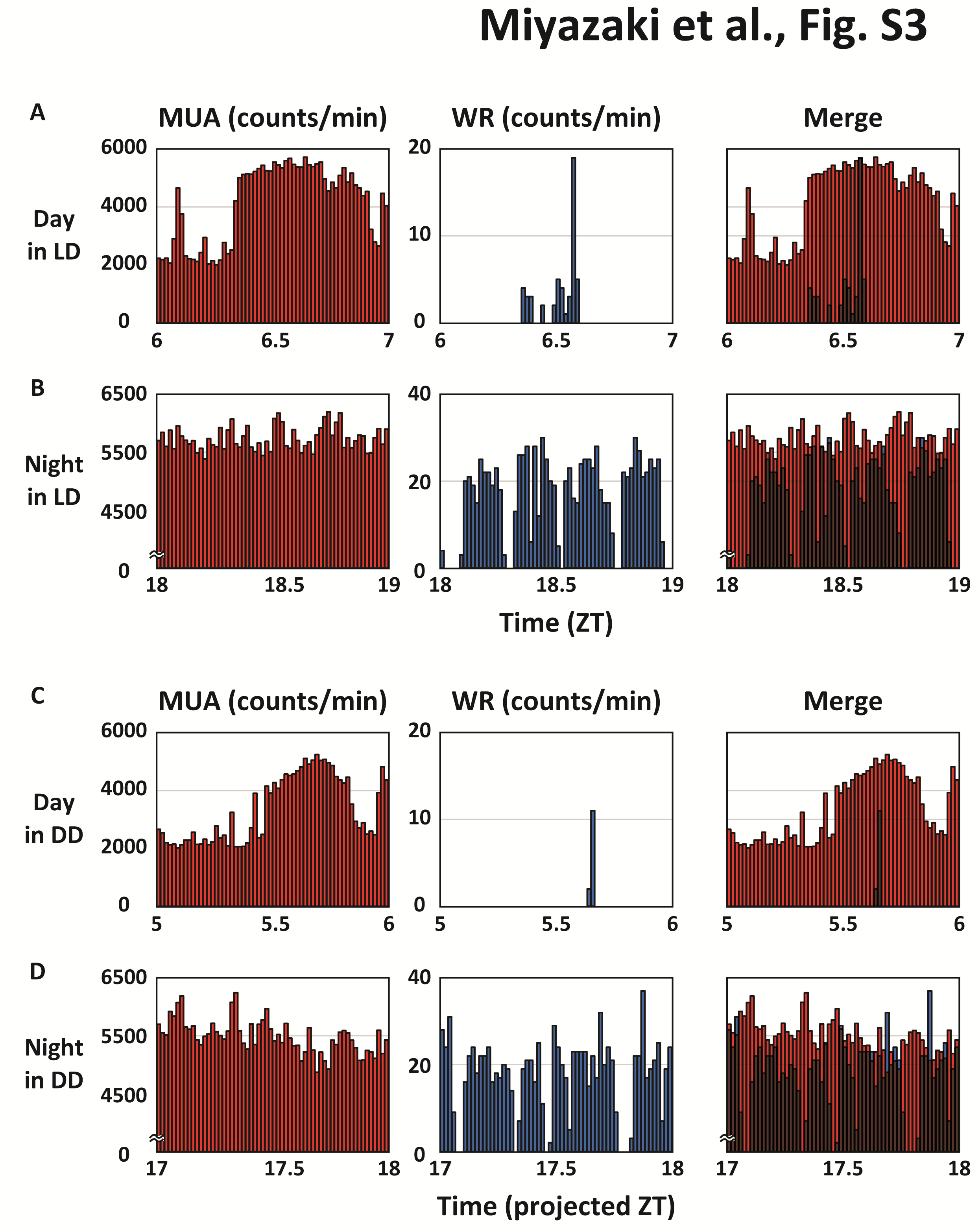


Enlarged actograms on the third day in the LD cycle or DD condition of Fig. 1B showing MUA in the striatum and WR for 1-hour in objective day (A) and night (B), or in subjective day (C) and night (D). A block means MUA and WR counts in 1 minute.

**Fig. S4 Circadian WR activity rhythms of METH-treated SCNi and SCNx mice.**


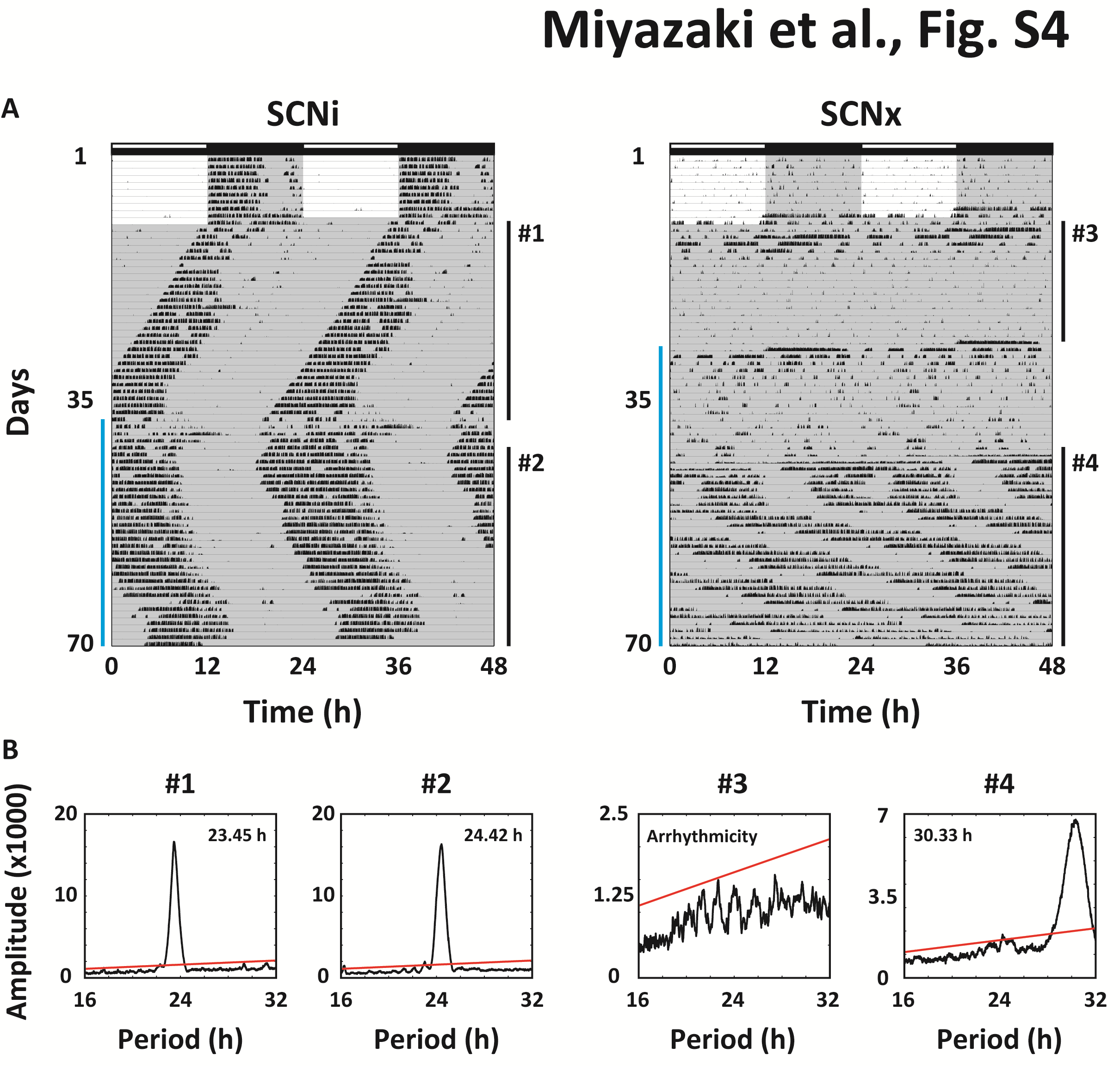


(A) Representative actograms showing diurnal and circadian rhythms of WR in METH-treated SCNi and SCNx mice. The SCNi mouse was exposure to 0.005 % METH dissolved in drinking water, after showing normal free-running activity in DD condition. As in the SCNi mouse, the SCNx mouse was exposure to 0.005 % METH dissolved in drinking water, after showing arrhythmic free-running activity in DD condition. The blue vertical line on the left side of actograms indicates the term of free access to 0.005 % METH dissolved in drinking water. (B) The chi-squared periodgram of WR rhythms in METH-treated SCNi and SCNx mice. The period was calculated according to the term of the black line on the right side of actograms (#1 ~ #4). The oblique red line indicates the significance level (**p* < 0.001).


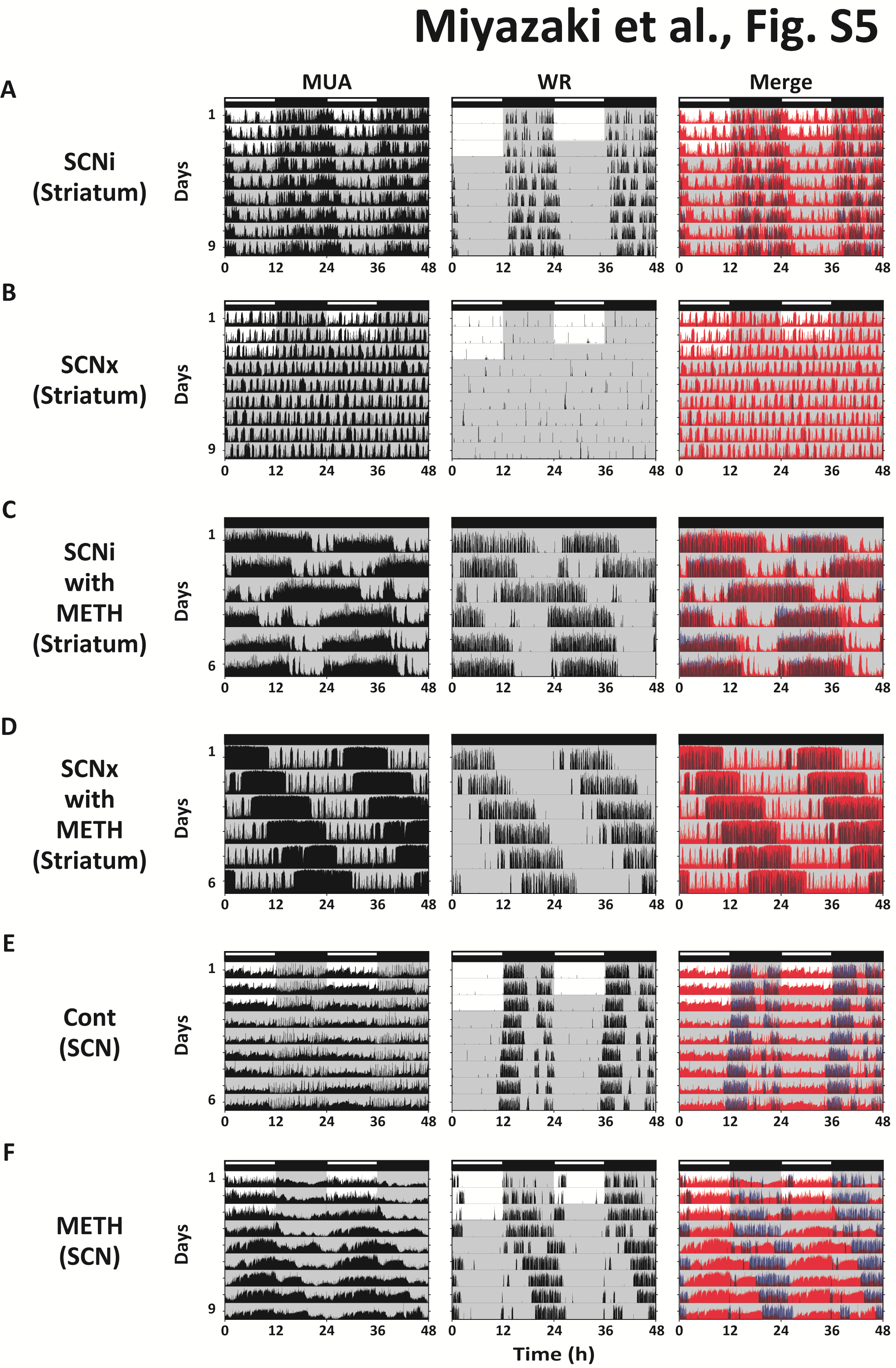


**Fig. S5 Another representative double-plotted actograms for experiment 1 – 3.**

(A, B) Representative double-plotted actograms showing diurnal and circadian rhythms of MUA in the striatum, WR and merged in SCNi (A) and SCNx (B) mice. The mice were maintained in the LD cycle and then transferred into the DD condition. (C, D) Representative double-plotted actograms showing circadian rhythms of MUA in the striatum, WR and merged in METH-treated SCNi (C) and SCNx mice (D). The actograms represent DD conditions for 6 days. (E, F) Representative actograms showing diurnal and circadian rhythms of MUA in the SCN, WR and merged in METH-untreated (E: Cont) and METH-treated (F: METH) mice. The mice were maintained in the LD cycle and then transferred into the DD condition. The red and blue actograms indicate MUA and WR, respectively. Lighting conditions are indicated at the top of the figure; open bars indicate a light phase and closed indicate a dark phase.
